# A transdiagnostic comparison of enhanced cognitive behaviour therapy (CBT-E) and interpersonal psychotherapy in the treatment of eating disorders

**DOI:** 10.1016/j.brat.2015.04.010

**Published:** 2015-07

**Authors:** Christopher G. Fairburn, Suzanne Bailey-Straebler, Shawnee Basden, Helen A. Doll, Rebecca Jones, Rebecca Murphy, Marianne E. O'Connor, Zafra Cooper

**Affiliations:** a Oxford University, Department of Psychiatry, UK; b Department of Population Health and Primary Care, Norwich Medical School, University of East Anglia, UK; c London School of Hygiene and Tropical Medicine, UK

**Keywords:** Eating disorders, Treatment, Cognitive behaviour therapy, Interpersonal psychotherapy

## Abstract

Eating disorders may be viewed from a transdiagnostic perspective and there is evidence supporting a transdiagnostic form of cognitive behaviour therapy (CBT-E). The aim of the present study was to compare CBT-E with interpersonal psychotherapy (IPT), a leading alternative treatment for adults with an eating disorder. One hundred and thirty patients with any form of eating disorder (body mass index >17.5 to <40.0) were randomized to either CBT-E or IPT. Both treatments involved 20 sessions over 20 weeks followed by a 60-week closed follow-up period. Outcome was measured by independent blinded assessors. Twenty-nine participants (22.3%) did not complete treatment or were withdrawn. At post-treatment 65.5% of the CBT-E participants met criteria for remission compared with 33.3% of the IPT participants (p < 0.001). Over follow-up the proportion of participants meeting criteria for remission increased, particularly in the IPT condition, but the CBT-E remission rate remained higher (CBT-E 69.4%, IPT 49.0%; p = 0.028). The response to CBT-E was very similar to that observed in an earlier study. The findings indicate that CBT-E is potent treatment for the majority of outpatients with an eating disorder. IPT remains an alternative to CBT-E, but the response is less pronounced and slower to be expressed.

**Current controlled trials:**

ISRCTN 15562271.

## Highlights

• CBT-E had a substantial and sustained effect.
• The effect was seen across the entire transdiagnostic sample.
• The response almost exactly replicated that obtained in the original CBT-E trial.
• The response to interpersonal psychotherapy (IPT) was slower and less pronounced.

## Graphical abstract

**Figure:**
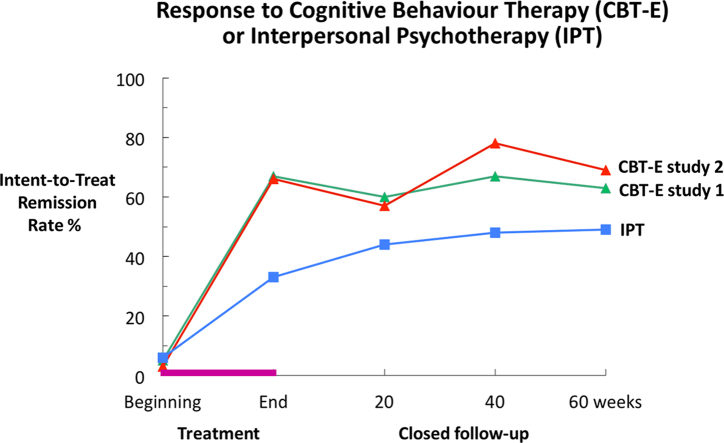

## 1 Introduction

Most studies of mental disorders have focused on specific disorders in isolation. This strategy has been criticised as it complicates the identification of psychopathological processes that operate across disorders (Insel, 2014; Insel et al., 2010) and it makes it difficult to identify treatments that are potentially “transdiagnostic” in their clinical range.

In 2003 we suggested that there would be value in viewing eating disorders from a “transdiagnostic” perspective (Fairburn, Cooper, & Shafran, 2003). We proposed that eating disorder psychopathology is maintained by a largely common set of mechanisms, and that treatments capable of addressing these mechanisms should be effective across the various eating disorder presentations. Accordingly, we modified the leading evidence-based treatment for the eating disorders, a cognitive behavioural treatment for bulimia nervosa (CBT-BN) (Fairburn, 1981; Fairburn, Marcus, & Wilson, 1993), to make it suitable for all forms of eating disorder while at the same time we attempted to make it more potent (Cooper & Fairburn, 2011; Fairburn et al., 2003). The resulting treatment, enhanced cognitive behaviour therapy or CBT-E, has been investigated as a treatment for anorexia nervosa (Dalle Grave, Calugi, Conti, Doll, & Fairburn, 2013; Dalle Grave, Calugi, Doll, & Fairburn, 2013; Dalle Grave, Calugi, Ghoch, Conti M, & Fairburn, 2014; Fairburn et al., 2013; Zipfel et al., 2014) and bulimia nervosa (Poulsen et al., 2014; Wonderlich et al., 2014), and in two transdiagnostic samples (Fairburn et al., 2009) that have included cases of bulimia nervosa, binge eating disorder and the various unspecified eating disorder presentations seen in adults (collectively termed “eating disorder not otherwise specified” in DSM-IV (American Psychiatric Association, 1994)). The findings indicate that CBT-E is indeed transdiagnostic in its clinical range, but its relative and absolute effectiveness remain to be established. Neither are clear because there have been differences across the studies in the way that CBT-E has been implemented.

The primary aim of the present study was to compare the effects of CBT-E with those of interpersonal psychotherapy (IPT), the leading alternative to cognitive behaviour therapy as a treatment for adult outpatients with an eating disorder. There is evidence supporting the use of IPT in bulimia nervosa (Agras, Walsh, Fairburn, Wilson, & Kraemer, 2000; Fairburn, Jones, Peveler, Hope, & O'Connor, 1993), binge eating disorder (Wilfley et al., 1993, 2002; Wilson, Wilfley, Agras, & Bryson, 2010) and, to a lesser extent, anorexia nervosa (Carter et al., 2011; McIntosh et al., 2005). IPT has not previously been tested in a broad transdiagnostic patient sample nor has it been compared with CBT-E. The second aim was to determine whether the findings of the original and largest study of CBT-E (Fairburn et al., 2009) could be replicated when an equivalent inclusive patient sample was recruited and CBT-E was implemented in the same way.

## 2 Method

### 2.1 Design

A randomized controlled trial was conducted at a catchment area-based eating disorder clinic in the UK. Eligible patients were randomized either to CBT-E or IPT delivered over 20 weeks. They were assessed before and after treatment and then entered a closed follow-up period during which they were assessed at 20, 40 and 60 weeks post-treatment. During the follow-up period they received no further treatment unless it was judged necessary on clinical grounds. The study was restricted to eating disorder patients whose body mass index (BMI) was over 17.5 and under 40.0. The study was approved by the local human subjects committee.

### 2.2 Recruitment

**Fig. 1:**
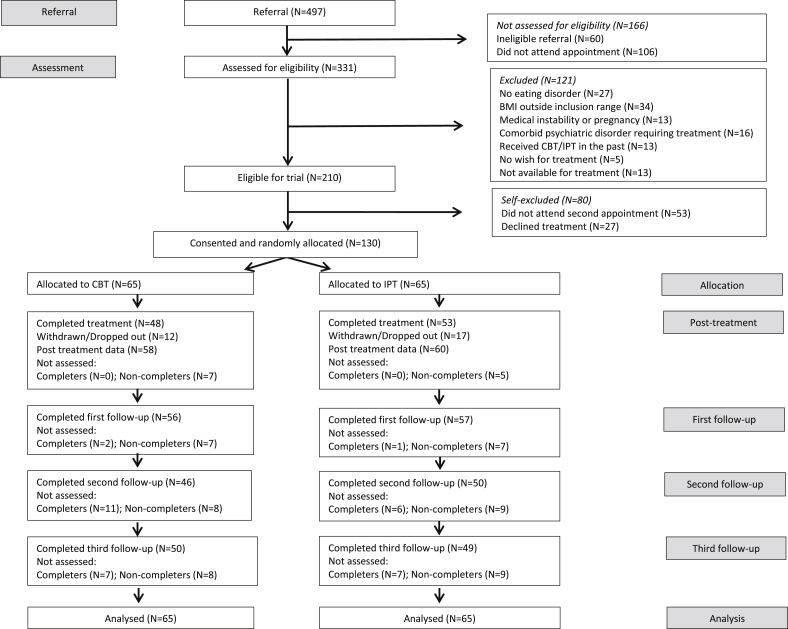
The CONSORT diagram.

The sample was recruited from consecutive referrals from family doctors and other clinicians to a long-established eating disorder clinic serving central Oxfordshire in the UK. Fig. 1 shows the CONSORT diagram.

Recruitment was designed to be “inclusive” with few exclusion criteria. The eligibility criteria were having an eating disorder that required treatment, as judged both by the referring clinician and, subsequently, by a senior eating disorder specialist (ZC or CGF); being aged 18–65 years; having a BMI over 17.5 and under 40.0; and providing written informed consent after receiving a detailed description of the study. The exclusion criteria were prior receipt of a treatment closely resembling CBT-E or IPT (N = 13); a co-existing general psychiatric disorder that precluded eating disorder-focused treatment (N = 16); medical instability or pregnancy (N = 13); and not being available for treatment (N = 13). Examples of co-existing general psychiatric disorders that precluded immediate entry into the trial were severe clinical depression, bipolar disorder and marked agoraphobia. Participants who were receiving ongoing psychiatric treatment were weaned off this prior to entering the study, the exception being clinically warranted antidepressant medication (N = 57) which was kept stable during treatment.

### 2.3 Treatment

Both treatments were delivered in 20 50-min sessions, preceded by one 90-min preparatory session, and followed by a review session 20 weeks after the completion of treatment.

*Enhanced cognitive behaviour therapy* – CBT-E is a psychological treatment designed to address eating disorder psychopathology whatever the eating disorder diagnosis. It is personalised to match the eating disorder psychopathology of the individual patient (Fairburn, 2008). The default (“focused”) form of the treatment was used. It concentrates exclusively on the modification of eating habits, weight-control behaviour, and concerns about eating, shape and weight.

*Interpersonal psychotherapy* – IPT is a short-term psychological treatment designed to identify and address current interpersonal problems (Klerman, Weissman, Rounsaville, & Chevron, 1984). It was originally developed as a treatment for depression but in the 1980s it was adapted by the first author to make it suitable for the treatment of bulimia nervosa (Fairburn, 1993). This form of IPT has been shown to be as effective as CBT-BN but it is substantially slower to achieve its results (Agras et al., 2000; Fairburn, Marcus, et al., 1993; Fairburn, Jones, et al., 1993). IPT has also been successfully used in the treatment of binge eating disorder (Wilfley et al., 2002; Wilson et al., 2010).

The form of IPT used in the present study has been described in detail elsewhere (Fairburn, 1993; Murphy, Straebler, Basden, Cooper, & Fairburn, 2012). It closely resembled IPT for depression but with minor adjustments so that it could be applied to people with any eating disorder (Murphy et al., 2012). It included all IPT's key strategies and procedures including role play.

*Therapists* – Three therapists took part, two were clinical psychologists and one was a psychiatric nurse practitioner. All had generic clinical experience and experience treating patients with eating disorders. One had prior experience implementing CBT-E and another had experience using IPT. Each therapist received six months' training from CGF and ZC in both CBT-E and IPT prior to the formal start of the study. During the study weekly supervision meetings were led by ZC and CGF with close and equal attention being paid to the quality of implementation of the two treatments. All the treatment sessions were recorded and each week selected sessions were audited by the supervisors.

*Treatment fidelity* – The quality of delivery of the two treatments was assessed by independent raters using an adaptation of an instrument developed for a prior comparison of CBT-BN and IPT (Loeb et al., 2005). There were 20 CBT-E items and these addressed general features of CBT-E, stage-specific features and the use of non-cognitive behavioural techniques. The 20 IPT items were equivalent in their focus but IPT-relevant. All the items were rated using the same seven-point rating scheme. Following completion of the treatment phase of the study 120 treatment sessions were selected at random from the early, middle, and late stages of both treatments and rated blind by four postgraduate research assistants who had received training in the use of the rating scale.

### 2.4 Assessment

*Eating disorder features* – These were assessed using the 16th edition of the Eating Disorder Examination interview (EDE) (Fairburn, Cooper, & O'Connor, 2008) and its self-report version (EDE-Q6.0) (Fairburn & Beglin, 2008). The EDE was administered by assessors who were trained and supervised by MO'C, an expert on the instrument. The assessors were kept blind to the participants' treatment condition and had no involvement with treatment. Operational DSM-IV diagnoses of bulimia nervosa and binge eating disorder (American Psychiatric Association, 1994) were generated from the EDE ratings (see Supplementary Table 1), the remaining participants being given the diagnosis “other eating disorder” (OED). Two outcome variables were created from the EDE ratings; the severity of eating disorder features as measured by the global EDE score (continuous), and the primary outcome variable of being in “remission” defined as a global EDE score less than 1 standard deviation (SD) above the community mean (categorical); i.e., below 1.74 (Beglin, 1990). Normative comparisons of this type are widely used to identify clinically significant change (Kazdin, 2003; Kendall, Marrs-Garcia, Nath, & Sheldrick, 1999).

*Psychosocial impairment* secondary to eating disorder features – This was measured using the Clinical Impairment Assessment questionnaire (CIA) (Bohn & Fairburn, 2008; Bohn et al., 2008).

*General psychiatric features* – The Structured Clinical Interview for DSM-IV (First, Gibbon, & Williams, 1997) was used at baseline to identify the presence of co-existing general psychiatric disorders, and the level of depressive features was measured using the Beck Depression Inventory (BDI) (Beck, Ward, Mendelson, Mock, & Erbaugh, 1961).

*Treatment suitability and expectancy* – These were assessed after two treatment sessions and suitability was assessed again at the end of treatment. Both were measured using widely employed patient-rated visual analogue scales (Agras et al., 2000).

### 2.5 Power and sample size

Sample size calculations were performed a priori with respect to the comparison of CBT-E and IPT, and they were on an intent-to-treat (ITT) basis. It was calculated that a sample size of 65 participants per treatment condition was required to provide 80% power at two-sided p < 0.05 to detect a difference at the end of treatment in global EDE change of 0.45 points, assuming a SD of global EDE change scores of 1.0 (Agras et al., 2000) (i.e., a moderate effect size of 0.5) and also to detect a difference between the two conditions of at least 25% in the categorical outcome measure.

### 2.6 Randomization

Participants were allocated to the two treatment conditions by HAD (who had no involvement in recruitment) using a computer-based minimization algorithm to balance on gender, eating disorder diagnosis, BMI, and the need for participants to remain on psychotropic medication. When groups were evenly balanced, pre-prepared blocked randomization lists of varying size were used to allocate participants to the two conditions.

### 2.7 Statistical methods

The primary analysis was an ITT analysis which included all randomised participants. Each continuous measure was analysed using a separate linear mixed model with the scores from each time point treated as a repeated measures outcome. Fixed effects of treatment (IPT vs CBT-E), time (baseline, end of treatment, and 20, 40 and 60 weeks post-treatment), and the interaction between treatment and time were specified, and the estimated baseline score was constrained to be identical in the two treatment conditions. This is equivalent to adjusting for baseline and permitting the relationship between baseline and follow up scores to differ at each time point, but offers the additional advantage that the data from all participants contribute to the analysis, even if there are missing data at follow up. An unstructured residual covariance matrix was used to allow for correlations within participants between the repeated measures over time. Each binary outcome was analysed in a similar fashion using a mixed effects logistic regression model with the binary indicator from each time point as the repeated measures outcome. Fixed effects were as for the continuous outcomes. A random effect of patient was specified to allow for correlations between repeated measures within participants. Statistical significance was taken at the 5% level (p < 0.05).

A per protocol analysis was also conducted using the same data analytic strategy as the ITT analysis. It was restricted to those participants who were considered by their therapist to have fully completed treatment.

All the analyses were undertaken by RJ using Stata v13 (StataCorp, College Station, Texas).

## 3 Results

### 3.1 Sample

One hundred and thirty eligible participants were recruited and randomized between 2006 and 2011. Their diagnoses were as follows: bulimia nervosa – 53 participants (40.8%); binge eating disorder – 8 participants (6.2%); and “other eating disorder” – 69 participants (53.1%). Sixty-five were randomized to CBT-E and 65 to IPT. The characteristics of the sample are shown in Table 1.

### 3.2 Suitability, expectancy, therapy quality and attrition

The ratings of treatment expectancy were high and did not differ between the two treatments (expectancy, mean and SD – IPT 66.1, 18.6; CBT-E 68.1, 20.5). The same was true of treatment suitability (beginning, mean and SD – IPT 69.2, 18.7; CBT-E 76.6, 19.7; end, mean and SD – IPT 75.5, 21.3; CBT-E 78.2, 24.4). The ratings of treatment fidelity were also high with over two-thirds of the sessions in both treatments being rated as “excellent” (i.e., ratings of 6 or 7 on the 1 to 7 point rating scale).

Twenty-nine participants (22.3%) did not complete all 20 sessions of treatment or were withdrawn because of lack of response. The non-completion figures by diagnosis were as follows: bulimia nervosa – 32.1% (17/53); binge eating disorder – 0% (0/8); other eating disorder – 17.4% (12/69).

### 3.3 Relative effects of CBT-E and IPT

#### 3.3.1 At post-treatment

**Fig. 2:**
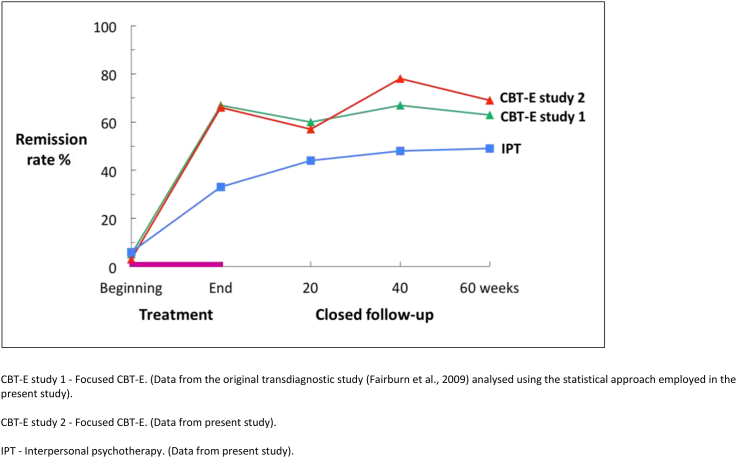
Intent-to-treat remission rates in the present study and the earlier one (Fairburn et al., 2009).

At post-treatment the levels of eating disorder and general psychopathology had decreased in both treatment conditions but the changes were significantly greater among the CBT-E participants (see Table 2 and Fig. 2). The observed proportion of CBT-E participants in remission (i.e., a global EDE score below 1.74) was almost twice that of those who received IPT (CBT-E − 38/58, 65.5%; IPT – 20/60, 33.3%; adjusted OR 8.8; 95% CI 2.6 to 29.5; p < 0.001; see Table 3). Almost half of the CBT-E participants (44.8%, 26/58) reported no binge eating, vomiting or laxative misuse at the end of treatment compared with 21.7% (13/60) in the IPT condition (adjusted OR 6.7; 95% CI 1.9 to 23.6; p = 0.003).

The changes observed were greater among the participants who completed treatment but the relative effects of the two treatments were similar (see Supplementary Tables 1 and 2). Three-quarters of those who completed CBT-E were in remission compared with just over a third of those who completed IPT (CBT-E − 36/48, 75.0%; IPT – 20/53, 37.7%; adjusted OR 13.0; 95% CI 3.4 to 49.4; p < 0.001; see Table 3).

#### 3.3.2 Over the 60-week post-treatment follow-up

There was high compliance with the 60-week period of post-treatment follow-up with 79.2% (309/390) of the assessments being successfully completed. Few of the participants required additional treatment. Of the 118 participants who were assessed at the end of treatment and then entered follow-up, seven had further treatment at some point in the 60 weeks and a further seven had up to five brief “booster” sessions.

The changes observed during treatment were well maintained in the CBT-E condition (see Table 2 and Fig. 2). For example, at the end of treatment the mean global EDE score was 1.57 (SD = 1.25) and at 60-week follow-up it was 1.51 (SD = 1.20). Over the same period the proportion of participants in remission increased slightly from 65.5% (38/58) at the end of treatment to 69.4% (34/49) at 60-week follow-up (see Fig. 2).

In the IPT condition the level of psychopathology fell over the period of follow-up with the result that many of the post-treatment differences between CBT-E and IPT were no longer statistically significant at 60-week follow-up (e.g. adjusted mean difference in global EDE score −0.28, 95% CI -0.74 to 0.18, p = 0.23; see Table 3), an exception being the primary outcome variable of being in remission which remained higher among the CBT-E participants than among those who received IPT (CBT-E − 34/49, 69.4%; IPT – 24/49, 49.0%; adjusted OR: 4.2; 95% CI: 1.2 to 15.1; p = 0.028; see Table 3 and Fig. 2).

The remission rates during the period of follow-up were higher among those who completed treatment but the relative effects of the two treatments remained similar (see Supplementary Tables 2 and 3). The proportion of participants in remission at 60-week follow-up was 70.0% (28/40) in the CBT-E condition and 50.0% (23/46) among those who received IPT (adjusted OR 3.8; 95% CI 1.0 to 14.6; p = 0.049).

### 3.4 Absolute effects of CBT-E

Fig. 2 shows the proportion of participants at each assessment point with a global EDE-Q score below 1.74, together with the equivalent data for CBT-E (focused version) from the earlier study (Fairburn et al., 2009) analysed using the statistical approach described above. It can be seen that in both studies the effects of CBT-E were substantial and well maintained. Furthermore, the magnitude of the response to CBT-E was remarkably similar in the two studies; the remission rates at the end of treatment being 67% in study 1 and 66% in study 2, and at 60-week post-treatment follow-up 63% and 69% respectively.

## 4 Discussion

This is the second randomised controlled trial to focus on the effects of CBT-E in a broad transdiagnostic sample of adult outpatients with an eating disorder (BMI >17.5 to <40.0). The first study recruited patients from two well-established eating disorder clinics in England, one in Leicestershire and the other in Oxfordshire (Fairburn et al., 2009). Both receive the great majority of referrals from the surrounding rural and urban areas. The present study recruited from the Oxfordshire-based clinic alone. In both studies care was taken to ensure that the great majority of referrals with a sufficiently severe eating disorder were eligible to take part. The resulting participant groups were very similar to each other. Both included the majority of those who were suitable and willing to be treated as outpatients, and they comprised patients with bulimia nervosa, binge eating disorder, the many and varied unspecified eating disorder presentations (Fairburn et al., 2007), and less underweight cases of anorexia nervosa (BMI > 17.5).

The first study compared the focused and broad forms of CBT-E, and it included a delayed treatment comparison condition (Fairburn et al., 2009). The present study involved a comparison of the focused form of CBT-E (the default version) with IPT, the leading alternative psychological treatment for adults with an eating disorder. Great care was taken to avoid any possible allegiance effects although none was likely as both treatments were devised by our group and we are known to be advocates of both CBT-E and IPT for eating disorders, The therapists received equivalent amounts of training in the two treatments and equivalent emphasis was placed upon them in the weekly supervision meetings. The independent ratings of treatment fidelity indicated that both were well implemented and there was no difference between the treatments in the ratings of suitability and expectancy.

The main aim of the study was to determine the relative effects of CBT-E and IPT. It was found that two-thirds of those who received CBT-E were in remission at the end of treatment compared with one-third of those who received IPT (CBT-E 66%; IPT 33%). This difference between the two treatments remained statistically significant 60 weeks later despite significant additional improvement in the IPT condition (CBT-E 69%; IPT 49%). The different temporal pattern of response to the two treatments (shown in Fig. 2) resembles that seen in the earlier studies of bulimia nervosa (Agras et al., 2000; Fairburn, Marcus, et al., 1993; Fairburn, Jones, et al., 1993). It is perhaps not surprising that CBT-E operates more rapidly than IPT given that it directly addresses the eating disorder psychopathology whereas IPT is likely to produce change indirectly via improvements in interpersonal functioning.

The second aim was to see whether the findings of the original and largest study of CBT-E (Fairburn et al., 2009) would be replicated when an equivalent patient sample was recruited and CBT-E was implemented in a consistent way following the treatment guide. It emerged that CBT-E had a remarkably similar effect (see Fig. 2). Using the statistical approach described above, two-thirds of the participants in both studies were in remission by the end of treatment, despite an average duration of eating disorder at presentation of 8.1 years in the original study (Fairburn et al., 2009) and 8.4 years in the present one. Furthermore in both studies the changes were well maintained. Sixty-three percent of the participants in the original study and 69% in the present one were in remission at the end of the 60-week closed period of follow-up yet there was minimal exposure to additional treatment.

A question of great importance is whether the effects seen in these RCTs are mirrored in “real world” clinical settings. In such settings the patients may be more heterogeneous; there may be a waiting list for treatment; and the therapists generally receive limited training and supervision. With regard to patient characteristics, Wales and colleagues showed that the Leicestershire RCT sample was extremely similar to the patients routinely seen in the same long-established clinic (Wales, Palmer, & Fairburn, 2009). Three studies have decribed the response rates obtained in real world settings. Byrne and colleagues in Australia reported the outcome of 125 consecutive patients treated with CBT-E (Byrne, Fursland, Allen, & Watson, 2011) and Knott and colleagues in a National Health Service (NHS) clinic in Wales described the response of a further 272 patients (Knott, Woodward, Hoefkens, & Limbert, 2015). In a third report Turner and colleagues (Turner, Marshall, Stopa, & Waller, 2015) described the outcome of 203 patients attending an NHS clinic in England, all of whom received an amalgam of CBT-E and the overlapping approach of Waller and colleagues (Waller et al., 2007). In all three cohorts (comprising a total of 600 patients) the outcome of the patients who completed CBT-E was similar to that reported here and in the original 2009 CBT-E trial. The main difference was that the treatment completion rates were lower. It therefore seems that CBT-E can be successfully used in routine clinical settings, the main challenge being to enhance patient retention. We are not aware of equivalent “real world” data on the use of IPT to treat eating disorders.

The present study had three significant strengths. First, like its predecessor, a clinically relevant sample was recruited from a long-established clinic using “inclusive” eligibility criteria. Second, the sample was transdiagnostic in composition. The great majority of adult outpatients with an eating disorder were represented. Third, we took pains to ensure that both treatments were implemented well and according to their respective protocols.

Five provisos must also be noted. First, this was a study of adult participants. It cannot be assumed that the same findings would be obtained with younger patients. CBT-E has been studied as a treatment for adolescents with anorexia nervosa (Dalle Grave, Calugi, Conti, et al., 2013; Dalle Grave, Calugi, Doll, et al., 2013; Dalle Grave, Calugi, Ghoch, Conti, & Fairburn, 2014) and the results appear promising but it has not been compared with other treatments for this age group nor has data been published on its use with adolescents with other eating disorder diagnoses. Second, as the study was restricted to participants with a BMI over 17.5 and under 40.0, it would not be appropriate to generalise the findings to patients with a BMI outside this range. This is important to note while keeping in mind the fact that the majority of adult outpatients with an eating disorder have a BMI within the range studied. Third, the duration of post-treatment follow-up was just 60 weeks long. While this covers the time of greatest risk of relapse, a longer period of follow-up would be of interest and is underway. Fourth, only the focused form of CBT-E was studied: the more complex “broad” form was not used. This decision was taken as there is evidence that it is no more effective overall than the focused form (Fairburn et al., 2009) yet it is more difficult to deliver. Furthermore, as the “interpersonal module” of broad CBT-E (Fairburn, 2008) overlaps substantially with IPT in its strategies and procedures, to have included it in the design would have confounded the comparison. Lastly, we have not reported on mediators or moderators of response to the two treatments. In separate publications we will examine whether there are moderators of response to CBT-E and IPT. We will also test specific mediational hypotheses concerning how both CBT-E and IPT operate using detailed within-treatment data collected for this purpose (Murphy, Cooper, Hollon, & Fairburn, 2009).

The findings presented here, taken together with those from the earlier study (Fairburn et al., 2009), indicate that CBT-E is a potent treatment that can be used with the majority of adult outpatients with an eating disorder; namely, those with a BMI over 17.5 and under 40.0. This broad clinical range makes the treatment of considerable value to clinicians. The findings also suggest that IPT has a broad clinical range although the response is slower to be expressed and is less pronounced. Lastly, the results underscore the value of research on transdiagnostic participant samples, as advocated by the RDoC initiative (Insel, 2014; Insel et al., 2010), as findings of this nature could not emerge from single-diagnosis research designs.

## Conflicts of interest

None.

## Figures and Tables

**Table 1:** Characteristics of the sample at baseline.

	IPT (N = 65)		CBT-E (N = 65)		All Patients (N = 130)	
	**Mean**	**(SD)**	**Mean**	**(SD)**	**Mean**	**(SD)**
Age (years)	26.8	(8.8)	24.9	(6.4)	25.9	(7.7)
	**N**	**(%)**	**N**	**(%)**	**N**	**(%)**
Female	63	(96.9)	64	(98.5)	127	(97.7)
Ethnicity:						
White	60	(92.3)	64	(98.5)	124	(95.4)
Black British	1	(1.5)	0	(0.0)	1	(0.8)
Asian Chinese	0	(0.0)	1	(1.5)	1	(0.8)
Asian British	1	(1.5)	0	(0.0)	1	(0.8)
Mixed	3	(4.6)	0	(0.0)	3	(2.3)
Marital Status:						
Single, never married	52	(80.0)	58	(89.2)	110	(84.6)
Married or living as such	12	(18.5)	6	(9.2)	18	(13.9)
Separated or divorced	1	(1.5)	1	(1.5)	2	(1.5)
Occupational Social Class:						
Higher	18	(27.7)	8	(12.3)	26	(20.0)
Intermediate	5	(7.7)	9	(13.9)	14	(10.8)
Lower	10	(15.4)	11	(16.9)	21	(16.2)
Unclassifiable	2	(3.1)	2	(3.1)	4	(3.1)
Student	30	(46.2)	35	(53.9)	65	(50.0)
DSM-IV Eating Disorder Status:						
Bulimia nervosa	28	(43.1)	25	(38.5)	53	(40.8)
Binge eating disorder	4	(6.2)	4	(6.2)	8	(6.2)
Other eating disorder	33	(50.8)	36	(55.4)	69	(53.1)
History of anorexia nervosa	15	(23.8)	21	(32.8)	36	(28.4)
	**Mean**	**(SD)**	**Mean**	**(SD)**	**Mean**	**(SD)**
Duration of eating disorder (years)	11.4	(9.6)	8.4	(7.3)	9.9	(8.6)
Lowest adult BMI (kg/m^2^)	19.3	(2.9)	18.9	(2.8)	19.1	(2.9)
Highest adult BMI (kg/m^2^)	26.6	(5.6)	26.2	(5.4)	26.4	(5.5)
	**N**	**(%)**	**N**	**(%)**	**N**	**(%)**
Current Comorbid Diagnoses (SCID):						
Major depressive episode	6	(9.2)	9	(13.9)	15	(11.5)
Any anxiety disorder	15	(23.1)	14	(21.5)	29	(22.3)
Substance abuse	7	(10.8)	2	(3.1)	9	(6.9)
Any Axis 1 disorder	25	(38.5)	22	(34.4)	47	(36.4)
Need for psychotropic medication	27	(41.5)	30	(46.2)	57	(43.9)

BMI – Body mass index; SCID – Structured Clinical Interview for DSM-IV Axis I Disorders (First et al., 1997). Missing values – There were 2 missing values for history of anorexia nervosa (IPT: 1 missing; CBT: 1 missing) and 1 missing value for any axis I disorder (IPT: 0 missing; CBT: 1 missing). The denominator for percentages is the number of non-missing values.

**Table 2:** Main clinical features at baseline, post-treatment and at 60-week post-treatment follow-up.

	Baseline				Post-treatment				60-week Follow-up			
	IPT (N = 65)		CBT (N = 65)		IPT (N = 60)		CBT (N = 58)		IPT (N = 49)		CBT (N = 49)	
	**Mean**	**(SD)**	**Mean**	**(SD)**	**Mean**	**(SD)**	**Mean**	**(SD)**	**Mean**	**(SD)**	**Mean**	**(SD)**
Body mass index (kg/m^2^)	22.8	(4.2)	22.9	(4.4)	23.6	(4.5)	23.5	(4.1)	24.8	(5.1)	24.1	(4.8)
Eating disorder psychopathology (EDE)												
Global score	3.52	(1.05)	3.59	(1.01)	2.37	(1.25)	1.57	(1.25)	1.83	(1.28)	1.51	(1.20)
Dietary restraint	3.70	(1.32)	3.71	(1.09)	2.46	(1.61)	1.08	(1.35)	1.71	(1.53)	1.32	(1.53)
Eating concern	2.86	(1.11)	2.81	(1.27)	1.83	(1.40)	1.12	(1.21)	1.24	(1.23)	0.97	(1.09)
Shape concern	4.03	(1.32)	4.08	(1.38)	2.53	(1.34)	1.99	(1.43)	2.30	(1.57)	1.98	(1.48)
Weight concern	3.47	(1.51)	3.77	(1.31)	2.67	(1.65)	2.09	(1.67)	2.08	(1.60)	1.78	(1.41)
Other features												
Secondary impairment (CIA)	30.0	(8.3)	30.5	(8.6)	19.6	(12.4)	13.9	(10.4)	12.6	(10.8)	12.4	(12.1)
Depressive features (BDI)	22.8	(10.9)	21.2	(10.8)	14.0	(12.3)	11.8	(11.0)	11.8	(12.6)	12.7	(11.8)
	**N**	**(%)**	**N**	**(%)**	**N**	**(%)**	**N**	**(%)**	**N**	**(%)**	**N**	**(%)**
EDE global score < 1 SD above the community mean (<1.74)	4	(6.2)	2	(3.1)	20	(33.3)	38	(65.5)	24	(49.0)	34	(69.4)
Eating disorder behaviour (EDE)												
Objective bulimic episodes ≥1	51	(78.5)	54	(83.1)	38	(63.3)	25	(43.1)	20	(40.8)	19	(38.8)
Self-induced vomiting ≥1	42	(64.6)	41	(63.1)	31	(51.7)	22	(37.9)	19	(38.8)	19	(38.8)
Laxative-taking ≥1	17	(26.2)	7	(10.8)	12	(20.0)	1	(1.7)	5	(10.2)	1	(2.0)
Absence of all of the above	8	(12.3)	6	(9.2)	13	(21.7)	26	(44.8)	23	(46.9)	22	(44.9)
Cessation of binge eating and purging if present at baseline (N, %)	–	–	–	–	7/52	(13.5)	22/53	(41.5)	16/41	(39.0)	18/45	(40.0)
	**Median**	**(IQR)**	**Median**	**(IQR)**	**Median**	**(IQR)**	**Median**	**(IQR)**	**Median**	**(IQR)**	**Median**	**(IQR)**
Objective bulimic episodes (N)	14	(3, 28)	11	(4, 28)	7	(0, 18)	0	(0, 5)	0	(0, 4)	0	(0, 2)
Self-induced vomiting (N)	14	(0, 40)	5	(0, 25)	1.5	(0, 19)	0	(0, 5)	0	(0, 7)	0	(0, 3)
Laxative-taking (N)	0	(0, 1)	0	(0, 0)	0	(0, 0)	0	(0, 0)	0	(0, 0)	0	(0, 0)

EDE – Eating Disorder Examination (Fairburn et al., 2008); CIA – Clinical Impairment Assessment (Bohn & Fairburn, 2008; Bohn et al., 2008); BDI – Beck Depression Inventory (Beck et al., 1961); BMI – Body mass index. Missing values – Immediately post-treatment there were a further 4 missing values for BMI, CIA and BDI (IPT: 1; CBT: 3). At 60-week post-treatment, there were a further 6 missing values for BMI (IPT: 4; CBT: 2), 14 missing for CIA (IPT: 7; CBT: 7) and 10 missing for BDI (IPT: 4; CBT: 6).

**Table 3:** Intention-to-treat analysis at post-treatment and at 60-week post-treatment follow-up.

	Effect estimates for CBT-E vs IPT post-treatment			Effect estimate for CBT-E vs IPT 60-week follow-up		
	**Difference**	**(95% CI)**	**p value**	**Difference**	**(95% CI)**	**p value**
Body mass index (kg/m^2^)	0.14	(−0.42–0.71)	0.621	−0.59	(−1.50–0.32)	0.204
Eating disorder psychopathology (EDE)						
Global score	−0.81	(−1.23–−0.40)	<0.001	−0.28	(−0.74–0.18)	0.230
Dietary restraint	−1.39	(−1.89–−0.89)	<0.001	−0.42	(−0.98–0.14)	0.142
Eating concern	−0.69	(−1.14–−0.23)	0.003	−0.23	(−0.68–0.21)	0.304
Shape concern	−0.54	(−0.99–−0.08)	0.021	−0.29	(−0.85–0.26)	0.303
Weight concern	−0.66	(−1.21–−0.11)	0.019	−0.28	(−0.82–0.26)	0.308
Other features						
Secondary Impairment (CIA)	−6.22	(−10.10–−2.33)	0.002	−1.33	(−5.72–3.05)	0.551
Depressive features (BDI)	−1.76	(−5.86–2.34)	0.399	1.12	(−3.40–5.63)	0.628
	**Odds ratio**	**(95% CI)**	**p value**	**Odds ratio**	**(95% CI)**	**p value**
EDE severity score < 1 SD above the community mean (<1.74)	8.75	(2.59–29.54)	<0.001	4.20	(1.17–15.14)	0.028
Eating disorder behaviour (EDE)						
Objective bulimic episodes ≥1	0.24	(0.08–0.75)	0.014	0.80	(0.24–2.66)	0.714
Self-induced vomiting ≥1	0.22	(0.04–1.09)	0.064	0.84	(0.15–4.79)	0.843
Laxative-taking ≥1	0.03	(0.00–0.54)	0.018	0.11	(0.00–2.82)	0.183
Absence of all of the above	6.68	(1.89–23.59)	0.003	1.04	(0.29–3.67)	0.954

EDE – Eating Disorder Examination (Fairburn et al., 2008); CIA – Clinical Impairment Assessment (Bohn & Fairburn, 2008; Bohn et al., 2008); BDI – Beck Depression Inventory (Beck et al., 1961). Estimated treatment differences are from longitudinal mixed effects linear or logistic regression models which included data from all participants who were randomised (IPT: 65; CBT: 65), account for missing data and repeated measures on individuals over time and adjust for baseline values of the outcome in question.

## References

[bib1] Agras W., Walsh B., Fairburn C.G., Wilson G., Kraemer H. (2000). A multicenter comparison of cognitive-behavioral therapy and interpersonal psychotherapy for bulimia nervosa. Archives of General Psychiatry.

[bib2] American Psychiatric Association (1994). Diagnostic and statistical manual of mental disorders.

[bib3] Beck A., Ward C., Mendelson M., Mock J., Erbaugh J. (1961). An inventory for measuring depression. Archives of General Psychiatry.

[bib4] Beglin S.J. (1990). Eating disorders in young adult women.

[bib5] Bohn K., Doll H.A., Cooper Z., O'Connor M., Palmer R.L., Fairburn C.G. (2008). The measurement of impairment due to eating disorder psychopathology. Behaviour Research and Therapy.

[bib6] Bohn K., Fairburn C.G., Fairburn C. (2008). Clinical impairment assessment questionnaire (CIA 3.0). Cognitive behavior therapy and eating disorders.

[bib7] Byrne S., Fursland A., Allen K., Watson H. (2011). The effectiveness of enhanced cognitive behavioural therapy for eating disorders: an open trial. Behaviour Research and Therapy.

[bib8] Carter F.A., Jordan J., McIntosh V.V.W., Luty S.E., McKenzie J.M., Frampton C.M.A. (2011). The long-term efficacy of three psychotherapies for anorexia nervosa: a randomized, controlled trial. International Journal of Eating Disorders.

[bib9] Cooper Z., Fairburn C.G. (2011). The evolution of “enhanced” cognitive behavior therapy for eating disorders: learning from treatment nonresponse. Cognitive and Behavioral Practice.

[bib10] Dalle Grave R., Calugi S., Conti M., Doll H., Fairburn C.G. (2013). Inpatient cognitive behaviour therapy for anorexia nervosa: a randomized controlled trial. Psychotherapy and Psychosomatics.

[bib11] Dalle Grave R., Calugi S., Doll H.A., Fairburn C.G. (2013). Enhanced cognitive behaviour therapy for adolescents with anorexia nervosa: an alternative to family therapy?. Behaviour Research and Therapy.

[bib12] Dalle Grave R., Calugi S., Ghoch M.E., Conti M., Fairburn C.G. (2014). Inpatient cognitive behavior therapy for adolescents with anorexia nervosa: immediate and longer-term effects. Frontiers in Psychiatry.

[bib13] Fairburn C.G. (1981). A cognitive behavioural approach to the treatment of bulimia. Psychological Medicine.

[bib14] Fairburn C.G., Klerman G.L., Weissman M.M. (1993). Interpersonal psychotherapy for bulimia nervosa. New applications of interpersonal psychotherapy.

[bib15] Fairburn C.G. (2008). Cognitive behavior therapy and eating disorders.

[bib16] Fairburn C., Beglin S.J., Fairburn C. (2008). Eating disorder examination questionnaire (EDE-Q 6.0). Cognitive behavior therapy and eating disorders.

[bib17] Fairburn C.G., Cooper Z., Bohn K., O'Connor M.E., Doll H.A., Palmer R.L. (2007). The severity and status of eating disorder NOS: Implications for DSM-V. Behaviour Research and Therapy.

[bib18] Fairburn C.G., Cooper Z., Doll H., O'Connor M., Bohn K., Hawker D. (2009). Transdiagnostic cognitive-behavioral therapy for patients with eating disorders: a two-site trial with 60-week follow-up. American Journal of Psychiatry.

[bib19] Fairburn C.G., Cooper Z., Doll H.A., O'Connor M.E., Palmer R.L., Dalle Grave R. (2013). Enhanced cognitive behaviour therapy for adults with anorexia nervosa: a UK-Italy study. Behaviour Research and Therapy.

[bib20] Fairburn C.G., Cooper Z., O'Connor M., Fairburn C. (2008). Eating disorder examination (Edition 16.0D). Cognitive behavior therapy and eating disorders.

[bib21] Fairburn C.G., Cooper Z., Shafran R. (2003). Cognitive behaviour therapy for eating disorders: a “transdiagnostic” theory and treatment. Behaviour Research and Therapy.

[bib22] Fairburn C.G., Jones R., Peveler R.C., Hope R.A., O'Connor M. (1993). Psychotherapy and bulimia nervosa. Longer-term effects of interpersonal psychotherapy, behavior therapy, and cognitive behavior therapy. Archives of General Psychiatry.

[bib23] Fairburn C.G., Marcus M.D., Wilson G.T., Fairburn C., Wilson G. (1993). Cognitive-behavioral therapy for binge eating and bulimia nervosa: a comprehensive treatment manual. Binge eating: Nature, assessment and treatment.

[bib24] First MB, RL, S., Gibbon, M., & Williams, J. (1997). User's guide for the Structured Clinical Interview for DSM-IV Axis I Disorders. Washington DC: American Psychiatric Press.

[bib25] Insel T. (2014). The NIMH research domain criteria (RDoC) project: precision medicine for psychiatry. American Journal of Psychiatry.

[bib26] Insel T., Cuthbert B., Garvey M., Heinssen R., Pine D.S., Quinn K. (2010). Research Domain Criteria (RDoC): toward a new classification framework for research on mental disorders. American Journal of Psychiatry.

[bib27] Kazdin A., Kazdin A. (2003). Clinical significance: measuring whether interventions make a difference. Methodological issues and strategies in clinical research.

[bib28] Kendall P., Marrs-Garcia A., Nath S., Sheldrick R. (1999). Normative comparisons for the evaluation of clinical significance. Journal of Consulting and Clinical Psychology.

[bib29] Klerman G.L., Weissman M.M., Rounsaville B.J., Chevron E.S. (1984). Interpersonal psychotherapy of depression.

[bib30] Knott S., Woodward D., Hoefkens A., Limbert C. (2015). Cognitive behaviour therapy for bulimia nervosa and eating disorders not otherwise specified: translation from randomized controlled trial to a clinical setting. Behavioural and Cognitive Psychotherapy.

[bib31] Loeb K.L., Wilson G.T., Labouvie E., Pratt E.M., Hayaki J., Walsh B.T. (2005). Therapeutic alliance and treatment adherence in two interventions for bulimia nervosa: a study of process and outcome. Journal of Consulting and Clinical Psychology.

[bib32] McIntosh V.V.W., Jordan J., Carter F.A., Luty S.E., McKenzie J.M., Bulik C.M. (2005). Three psychotherapies for anorexia nervosa: a randomized, controlled trial. American Journal of Psychiatry.

[bib33] Murphy R., Cooper Z., Hollon S.D., Fairburn C.G. (2009). How do psychological treatments work? Investigating mediators of change. Behaviour Research and Therapy.

[bib34] Murphy R., Straebler S., Basden S., Cooper Z., Fairburn C.G. (2012). Interpersonal psychotherapy for eating disorders. Clinical Psychology & Psychotherapy.

[bib35] Poulsen S., Lunn S., Daniel S., Folke S., Mathiesen B., Katznelson H. (2014). A randomized controlled trial of psychoanalytic psychotherapy or cognitive-behavioral therapy for bulimia nervosa. American Journal of Psychiatry.

[bib36] Turner H., Marshall E., Stopa L., Waller G. (2015). Cognitive-behavioural therapy for outpatients with eating disorders: effectiveness for a transdiagnostic group in a routine clinical setting. Behaviour Research and Therapy.

[bib37] Wales J., Palmer R.L., Fairburn C.G. (2009). Can treatment trial samples be representative?. Behaviour Research and Therapy.

[bib38] Waller G., Cordery H., Corstorphine E., Hinrichsen H., Lawson R., Mountford V. (2007). Cognitive behavioral therapy for eating disorders.

[bib39] Wilfley D.E., Agras W.S., Telch C.F., EM R., Schneider J., Golomb A. (1993). Group cognitive-behavioral therapy and group interpersonal psychotherapy for the nonpurging bulimic individual: a controlled comparison. Journal of Consulting and Clinical Psychology.

[bib40] Wilfley D.E., Welch R.R., Stein R.I., Spurrell E.B., Cohen L.R., Saelens B.E. (2002). A randomized comparison of group cognitive-behavioral therapy and group interpersonal psychotherapy for the treatment of overweight individuals with binge-eating disorder. Archives of General Psychiatry.

[bib41] Wilson G.T., Wilfley D.E., Agras W.S., Bryson S.W. (2010). Psychological treatments of binge eating disorder. Archives of General Psychiatry.

[bib42] Wonderlich S., Peterson C., Crosby R., Smith T., Klein M., Mitchell J. (2014). A randomized controlled comparison of integrative cognitive-affective therapy (ICAT) and enhanced cognitive-behavioral therapy (CBT-E) for bulimia nervosa. Psychological Medicine.

[bib43] Zipfel S., Wild B., Groß G., Friederich H., Teufel M., Schellberg D. (2014). Focal psychodynamic therapy, cognitive behaviour therapy, and optimised treatment as usual in outpatients with anorexia nervosa (ANTOP study): randomised controlled trial. The Lancet.

