# Supplementary material for: A transdiagnostic comparison of enhanced cognitive behaviour therapy (CBT-E) and interpersonal psychotherapy in the treatment of eating disorders
Source: Behav Res Ther. 2015 Jul;70:64–71. doi: 10.1016/j.brat.2015.04.010 (PMC4461007; doi:10.1016/j.brat.2015.04.010)
Supplement: Supplementary file 1 [file mmc1.pdf]

Supplementary Table 1. DSM-IV diagnostic criteria for eating disorders (American Psychiatric Association, 1994) and their EDE-based operational definitions (Fairburn et al., 2008).

DSM-IV diagnosis	DSM-IV diagnostic criteria	EDE-based operational definition
Anorexia Nervosa	A. Refusal to maintain one's body weight at or above a minimally normal weight for age and height	Body mass index below or equal to 17.5 and a rating of 1 on the EDE item "Maintained low weight".
	B. Intense fear of gaining weight or becoming fat, even though underweight.	A rating of higher or equal to 4 on the EDE item "Fear of weight gain" in each of the three months prior to assessment.
	C. Disturbance in the way in one's body weight or shape is experienced, undue influence of body weight or shape on self-evaluation, or denial of the seriousness of the current low body weight.	A rating of higher or equal to 4 on one or other of the EDE items "Importance of weight" and "Importance of shape" in each of the three months prior to assessment.
	D. Amenorrhea	A rating of 0 or 44 on the EDE item Menstruation
Bulimia Nervosa	A. Recurrent episodes of binge eating.	24 or more objective bulimic episodes within the three months prior to assessment and eight or more objective bulimic episodes in the four weeks prior to assessment.
	B. Recurrent inappropriate compensatory behavior in order to prevent weight gain, such as self-induced vomiting; misuse of laxatives, diuretics, enemas, or other medications; fasting; or excessive exercise.	24 or more episodes of either self-induced vomiting (SIV) or laxative misuse or diuretic misuse over past three months (i.e., total number of "purging" occasions over past 3 months higher or equal to 24), and 8 or more purging episodes in the four weeks prior

		to assessment.<br><br>Alternatively, extreme dietary restriction (i.e., fasting) outside bulimic episodes as assessed using the EDE item "Dietary restriction outside bulimic episodes" (rating of 1 or 2). NB: The definition does not utilise exercise as a diagnostic criterion for bulimia nervosa.
	C. The binge eating and inappropriate compensatory behaviour both occur, on average, at least twice a week for three months.	(Defined above)
	D. Self-evaluation is unduly influenced by body shape and weight.	A rating of higher or equal to 4 on one or other of the EDE items "Importance of weight" and "Importance of shape" in each of the three months prior to assessment.
	E. The disturbance does not occur exclusively during episodes of anorexia nervosa.	A diagnosis of anorexia nervosa is made if the diagnostic criteria for both bulimia nervosa and anorexia nervosa are fulfilled.<br>In addition, body mass index should be above 17.5.
Binge Eating Disorder	A. Recurrent episodes of binge-eating.	Objective bulimic episodes on at least 2 days a week on average over the six months prior to assessment and eight or more objective bulimic episodes in the four weeks prior to assessment.
	B. The binge-eating episodes are associated with three (or more) of the following:<br>(1) eating more rapidly than normal	A rating of 1 on three or more on these EDE Binge Eating Disorder module items.

(2) eating until feeling uncomfortably full<br>(3) eating large amounts of food when not feeling physically hungry<br>(4) eating alone because of being embarrassed by how much one is eating<br>(5) feeling disgusted with oneself, depressed, or very guilty after overeating.	
C. Marked distress regarding binge eating.	A rating of 1 on this EDE Binge Eating Disorder module item.
D. The binge eating occurs, on average, at least 2 days a week for 6 months.	(Defined above.)
E. The binge eating is not associated with the regular use of inappropriate compensatory behaviors (e.g. purging, fasting, excessive exercise) and does not occur exclusively during the course of Anorexia Nervosa or Bulimia Nervosa.	Absence of the regular use (i.e., three or more episodes per month) of compensatory behaviour (self-induced vomiting or laxative misuse or diuretic misuse or excessive exercise) over the past six months, and the absence of extreme dietary restriction (i.e., fasting) outside bulimic episodes, as assessed using the EDE item “Dietary restriction outside bulimic episodes” (rating of 1or 2).

Supplementary Table 2. Primary and secondary outcomes at baseline, immediately post treatment and 60 weeks post treatment in patients who completed treatment

	Baseline				Immediately post treatment				60 weeks post treatment			
	IPT (N = 53)		CBT (N = 48)		IPT (N = 53)		CBT (N = 48)		IPT (N = 46)		CBT (N = 40)	
	Mean	(SD)	Mean	(SD)	Mean	(SD)	Mean	(SD)	Mean	(SD)	Mean	(SD)
Body mass index (kg/m^2)	23.4	(4.4)	22.6	(4.2)	24.0	(4.5)	23.4	(4.1)	25.1	(5.2)	24.2	(4.9)
Eating disorder psychopathology (EDE)												
Global score	3.46	(1.05)	3.46	(0.96)	2.24	(1.22)	1.24	(0.91)	1.76	(1.22)	1.43	(1.16)
Dietary restraint	3.56	(1.21)	3.65	(1.17)	2.25	(1.58)	0.77	(1.00)	1.63	(1.46)	1.26	(1.54)
Eating concern	2.73	(1.10)	2.69	(1.19)	1.66	(1.31)	0.81	(0.82)	1.16	(1.15)	0.89	(1.01)
Shape concern	3.95	(1.38)	3.91	(1.33)	2.43	(1.36)	1.73	(1.29)	2.21	(1.51)	1.88	(1.47)
Weight concern	3.61	(1.50)	3.61	(1.30)	2.63	(1.66)	1.66	(1.34)	2.05	(1.59)	1.71	(1.38)
Other features												
Secondary impairment (CIA)	29.2	(8.2)	30.3	(9.2)	17.5	(11.2)	11.2	(7.6)	12.7	(10.9)	12.5	(12.7)
Depressive features (BDI)	21.6	(10.5)	19.8	(10.4)	11.7	(10.3)	8.2	(6.8)	11.2	(12.3)	12.2	(11.8)
	N	(%)	N	(%)	N	(%)	N	(%)	N	(%)	N	(%)
EDE global score < 1 SD above the community mean (<1.74)	4	(7.6)	1	(2.1)	20	(37.7)	36	(75.0)	23	(50.0)	28	(70.0)
Eating disorder behaviour (EDE)												
Objective bulimic episodes ≥1	42	(79.3)	41	(85.4)	33	(62.3)	22	(45.8)	18	(39.1)	14	(35.0)
Self-induced vomiting ≥1	31	(58.5)	29	(60.4)	25	(47.2)	17	(35.4)	17	(37.0)	15	(37.5)
Laxative-taking ≥1	12	(22.6)	6	(12.5)	8	(15.1)	1	(2.1)	5	(10.9)	0	(0.0)

Absence of all of the above	8	(15.1)	3	(6.3)	13	(24.5)	21	(43.8)	22	(47.8)	18	(45.0)
Cessation of all of the above, if present at baseline	–	–	–	–	7 / 45	(15.6)	19 / 45	(42.2)	15 / 38	(39.5)	15 / 37	(40.5)
	Median	(IQR)	Median	(IQR)	Median	(IQR)	Median	(IQR)	Median	(IQR)	Median	(IQR)
Eating disorder behaviour (EDE)												
Objective bulimic episodes (N)	10	(3, 27)	11.5	(5, 27)	5	(0, 18)	0	(0, 4.5)	0	(0, 4)	0	(0, 2)
Self-induced vomiting (N)	9	(0, 30)	3	(0, 26.5)	0	(0, 14)	0	(0, 2.5)	0	(0, 6)	0	(0, 3)
Laxative-taking (N)	0	(0, 0)	0	(0, 0)	0	(0, 0)	0	(0, 0)	0	(0, 0)	0	(0, 0)

EDE - Eating Disorder Examination (Fairburn et al., 2008); CIA - Clinical Impairment Assessment (Bohn & Fairburn, 2008; Bohn et al., 2008); BDI - Beck Depression Inventory (Beck et al., 1961).

Missing values - Immediately post treatment there were a further 2 missing values for BMI (IPT: 1; CBT: 1) and 3 missing values for CIA and BDI (IPT: 1; CBT: 2). At 60 weeks post treatment, there were a further 6 missing values for BMI (IPT: 4; CBT: 2), 11 missing values for CIA (IPT: 5; CBT: 6) and 8 missing values for BDI (IPT: 3; CBT: 5).

Supplementary Table 3. Per protocol analysis immediately post treatment and 60 weeks post treatment

	Effect estimates for CBT-E vs IPT immediately post treatment			Effect estimate for CBT-E vs IPT 60 weeks post treatment		
	Difference	(95% CI)	p value	Difference	(95% CI)	p value
Body mass index (kg/m^2)	0.02	(-0.52 to 0.55)	0.950	-0.42	(-1.35 to 0.50)	0.370
Eating disorder psychopathology (EDE)						
Global score	-1.00	(-1.40 to -0.60)	0.000	-0.30	(-0.78 to 0.18)	0.220
Dietary restraint	-1.50	(-2.00 to -1.01)	0.000	-0.42	(-1.01 to 0.17)	0.160
Eating concern	-0.84	(-1.27 to -0.41)	0.000	-0.25	(-0.71 to 0.20)	0.272
Shape concern	-0.69	(-1.16 to -0.21)	0.004	-0.31	(-0.91 to 0.28)	0.301
Weight concern	-0.97	(-1.52 to -0.43)	0.000	-0.29	(-0.87 to 0.28)	0.313
Other features						
Secondary impairment (CIA)	-7.14	(-10.77 to -3.51)	0.000	-1.67	(-6.38 to 3.04)	0.486
Depressive features (BDI)	-3.46	(-6.93 to 0.02)	0.051	0.89	(-3.84 to 5.61)	0.713
	Odds ratio	(95% CI)	p value	Odds ratio	(95% CI)	p value
EDE severity score < 1 SD above the community mean (<1.74)	13.02	(3.43 to 49.39)	0.000	3.83	(1.01 to 14.58)	0.049
Eating disorder behaviour (EDE)						
Objective bulimic episodes ≥1	0.32	(0.10 to 1.03)	0.057	0.83	(0.24 to 2.90)	0.767
Self-induced vomiting ≥1	0.23	(0.04 to 1.32)	0.099	1.13	(0.18 to 7.18)	0.896
Laxative-taking ≥1	–	–	–	–	–	–
Absence of all of the above	5.70	(1.48 to 21.93)	0.011	1.02	(0.27 to 3.91)	0.980

EDE - Eating Disorder Examination (Fairburn et al., 2008); CIA - Clinical Impairment Assessment (Bohn & Fairburn, 2008; Bohn et al., 2008); BDI - Beck Depression Inventory (Beck et al., 1961).

Estimated treatment differences are from longitudinal mixed effects linear or logistic regression models which included data from all participants who were randomised (IPT: 65; CBT: 65), account for missing data and repeated measures on individuals over time and adjust for baseline values of the outcome in question.
